# The Discrete Emotions Questionnaire: A New Tool for Measuring State Self-Reported Emotions

**DOI:** 10.1371/journal.pone.0159915

**Published:** 2016-08-08

**Authors:** Cindy Harmon-Jones, Brock Bastian, Eddie Harmon-Jones

**Affiliations:** 1 The University of New South Wales, Sydney, Australia; 2 University of Melbourne, Melbourne, Australia; University of Groningen, NETHERLANDS

## Abstract

Several discrete emotions have broad theoretical and empirical importance, as shown by converging evidence from diverse areas of psychology, including facial displays, developmental behaviors, and neuroscience. However, the measurement of these states has not progressed along with theory, such that when researchers measure subjectively experienced emotions, they commonly rely on scales assessing broad dimensions of affect (positivity and negativity), rather than discrete emotions. The current manuscript presents four studies that validate a new instrument, the Discrete Emotions Questionnaire (DEQ), that is sensitive to eight distinct state emotions: anger, disgust, fear, anxiety, sadness, happiness, relaxation, and desire. Emotion theory supporting the importance of distinguishing these specific emotions is reviewed.

## Introduction

Beginning with the theories of Darwin [1] and James [2], several theories of discrete emotions have been proposed [3, 4, 5, 6]. Evidence for sets of discrete emotions has been garnered from research on facial expressions and other behavioral expressions, as well as from direct brain stimulations of non-human animals. Surprisingly, these empirical advances have not been met with advances in the measurement of discrete emotions via self-report questionnaires. Instead, the dominant method of assessing self-reported affect is through questionnaires aimed at assessing broad categories of positive and negative affect. The research reported in this article aims to provide a new measure of discrete emotions that can be used to assess multiple state emotions. Doing so will aid the field by improving our ability to measure the subjective experience of emotions, which will aid in understanding not only emotional reactions but the emotional processes that underlie much of our cognitive and social processes and behavioral responses.

### On Accurately Assessing Self-Reported Emotions

Psychologists prefer to use established, published measures of constructs. As a result, many psychologists have chosen to use the Positive and Negative Affect Schedule (PANAS) [7] to measure self-reported positive and negative affect when testing whether their manipulation influenced emotions. Indeed, the PANAS, which was originally published in the *Journal of Personality and Social Psychology (JPSP)*, has been cited over 18,000 times (from Google Scholar, 22 July 2015), and is the most cited paper in *JPSP* behind Baron and Kenny’s [8] mediation-moderation paper (according to Google Scholar, 22 July 2015).

Often, research uses the PANAS to measure responses to situational manipulations of emotion, and often null effects are reported. Because emotions are only probabilistically linked to measures of emotion, it is inappropriate to assume that a null effect on a self-report measure means that emotional processes were not involved in the psychological phenomena of interest. That is, the (often poor) measurement of emotion should not be conflated with emotion itself. Emotion can be involved in driving a phenomenon despite null effects on measures of self-report.

Many psychological scientists use poor measures of emotion to discount emotion prematurely. In fact, we are scarcely immune from the allure of premature discounting, having done so in the not-so-distant past [9]. In this study, inspired by Terror Management Theory, participants were assigned to groups based on their ostensible painting preferences (minimal groups) and either prompted to write about their own death or watching television. They then completed the PANAS. We found no increase in negative affect or decrease in positive affect in the mortality salience condition, as measured by the PANAS, and argued that this suggested that affect was not involved in the effects of mortality salience. Similar flawed logic has been used in numerous published papers.

The way in which the field dismisses emotion based on self-report alone demonstrates the necessity for more comprehensive scrutiny of emotional processes in mainstream psychology. Emotion is a primary influence on psychological processes and behavior. It need not be controlled for; rather, it should always be considered and measured appropriately. Researchers do not administer cognitive measures in an attempt to “rule out” cognition as a factor in a psychological process (because cognition is assumed to play a role) and emotional/motivational processes are at least as fundamental as cognition. If an organism is behaving, it can safely be assumed it is motivated and emoting. Measuring emotion should be directed at identifying the contribution of emotion to the behavior.

As we have already alluded to, emotions involve more than self-reported feelings. Although assessing self-reported emotional responses may be appropriate (and accurate) in some cases, one of the most widely used measures of self-reported emotional experience, the PANAS [7], is fraught with problems that may lead to it failing to yield support for the role of affective processes as resulting from and being involved in psychological phenomena.

First, consider a bit of history about the development of the PANAS. When Watson and colleagues [7] developed it, they were intent on arriving at a measure that would provide statistically independent measures of positive versus negative affect, “that is, terms that had a substantial loading on one factor but a near-zero loading on the other” (p. 1064) [7]. So they began with a long list of emotion labels, and then discarded all words that did not fit the statistical independence of positive versus negative affect. Perhaps consequently, they arrived at a measure of positive affect that does not include many common positive emotion words such as *happy* or *joyful*, but instead includes items such as *alert*, *active*, and *attentive* that may not always be positive in valence (e.g., a person could be alert, active, and attentive during threat). Similarly, the measure of negative affect does not include many common negative emotion words such as *sad*, *down*, *grieved*, and *angry*. Watson and colleagues [10] later suggested that their measures should be referred to as positive activation and negative activation because the emotion words on the scale were activated (i.e., high arousal) positive and negative emotions. This is partly accurate and explains why sadness-related words are missing but it fails to explain why some positive affects are missing (e.g., *joyful*) and why less common activated negative affect words are used for some concepts (i.e., *irritated* is included, rather than *angry*).

It would seem that the PANAS may be limited only to those contexts where activated emotion states are involved, indicating the need for other validated measures of emotion. Yet, even the use of the PANAS in these contexts could be problematic. Firstly, recent factor analytic studies have revealed that the factor structure of the PANAS is not maintained under emotion-evoking manipulations. Instead, items load on additional factors reflecting discrete emotions. For example, from before, to during, to after taking an exam, the PA items clustered into three discrete positive emotions, “joy”, “interest”, and “activation” [11]. Whereas when participants were angered, PANAS items loaded on three factors: the PA items loaded on the first factor, the anger items on the second factor, and the fear/anxiety items on the third factor [12].

Another issue is that PA scores have been found to increase during anger (an approach-motivated state that is negative in valence), suggesting that the positive activation scale does not always measure positivity, but may be sensitive to approach motivation [12]. In support of the idea that PA reflects approach rather than pure positivity, recent research has revealed that the PANAS is contaminated by a large first factor that could be referred to as evaluation or social desirability [13]. This research has revealed that responses to PANAS positive activation words (e.g., proud) are highly positively correlated with responses to positive antonyms of those words (e.g., humble). When this first factor is statistically removed, anger correlates with positive activation, as revealed in other studies [12,14].

### Other Measures of Self-Reported Emotions

The PANAS is not the only way to measure self-reported emotions, yet other questionnaires are also commonly based on dimensional models, similar to the PANAS. Some of these questionnaires use multiple items to assess the broad dimensions of positive and negative affect, such as the Brief Mood Introspection Scale [15], or use single items to assess broad dimensions of valence, arousal, and dominance, such as the self-assessment manikin [16].

Some affect measures that do focus on discrete emotions have been developed for clinical purposes. The Profile of Mood States (POMS) [17] is a measure of emotion blends (i.e., anger-hostility, tension-anxiety, depression-dejection) and other affective states (e.g., fatigue-inertia; vigor-activity; confusion-bewilderment). It is a scale that must be purchased from the publisher in order to be used for research purposes. Since it was produced in 1971, the POMS has been used primarily in research and applied settings with psychiatric outpatients, medical patients, and in sports psychology. The Multiple Affect Adjective Checklist (MAACL) [18] and its revised version (MAACL-R) are comprised of subscales that are designed to assess self-reported Anxiety, Depression, Hostility, Positive Affect, and Sensation Seeking. Like the POMS, the MAACL has been used primarily in clinical psychology and psychiatry, and it has been used to assessed trait affect or day-to-day changes in affective states. Both the POMS and MAACL lack subscales for certain basic emotions (e. g., disgust). Furthermore, their usefulness in assessing the full range of emotional experience is limited by their focus on maladaptive affect.

Other studies examining situationally induced emotions have used single items to measure the discrete emotions evoked [19]. The use of single items to measure emotions has proven effective in these published studies, but generally speaking, methodologists express concerns over the use of single items to measure psychological constructs. Short measures are more likely to contain error variance [20] because they cannot benefit as much from aggregation across multiple items [21]. The use of a single item to measure a discrete emotion may not accurately capture an individual’s subjective emotional state because the individual may interpret the item differently at that moment.

In support, a review of personality questionnaires ranging from two to eight items in length found a positive correlation of r = .77 between questionnaire length and reliability [22]. If self-report questionnaires of emotions have low reliability, the chances of observing correlations between specific emotions and other measured constructs is reduced, because the correlation between two measures is limited by the reliability of each measure. In addition, because unreliable measures contain more measurement error, the determination of effect size between the unreliable measure and other measures will be more difficult than if reliable measures were used. Short measures of emotion, however, may prove to be more valid, as they assist in reducing participant boredom and fatigue [23]. Some research has suggested that the optimal trade-off between reliability and validity occurs with two to four items [24].

Other measures of discrete emotions have been produced, but these discrete emotions measures do not measure a larger set of emotions. For example, measures have been developed to assess the trait tendency to experience various discrete emotions, such as proneness to anger, awe, compassion, disgust, embarrassment, envy, gratitude, guilt and shame, happiness, and pride [25, 26, 27, 28, 29, 30, 31, 32, 33, 34]. However, these questionnaires measure traits and are not designed to measure state emotions.

Self-report questionnaires designed to assess state emotions exist for a few emotions, such as anxiety [35], pride [34], and shame and guilt [36]. But these state emotion questionnaires, like the trait emotion questionnaires, stand in isolation of other emotions and are not part of a questionnaire assessing a broad array of emotions. The current research was designed to provide a state measure of a wide array of emotions.

A single instrument that assesses discrete emotions relatively comprehensively is important for several reasons. One is that the same manipulation may evoke primarily one emotion in a particular individual, but a different emotion in another. For example, negative feedback on an essay might be intended to increase anger, and may in fact elevate anger in a majority of participants. However, if this manipulation evokes primarily sadness in a subset of participants, this would be missed if the researcher used only a measure of the single emotion of anger. Additionally, individuals may experience mixed emotions, which may have very different effects than the same emotion when not blended with other emotions. For example, anger is usually an approach-related emotion. However, when anger is blended with anxiety, it may be associated with withdrawal motivation [37]. A single instrument that assesses the broad range of emotions would allow researchers to identify when emotions are relatively pure versus when they are blended.

### Theories of Basic Emotions

In designing our Discrete Emotions Questionnaire (DEQ), we aimed to include emotions that are considered "basic" by prominent emotion theories. These theories of emotion were developed by Paul Ekman [3, 38], Carroll Izard [4, 5], Jaak Panksepp [6], and Phillip Shaver and colleagues [39]. Although none of these theorists consider their lists to encompass the entire range of possible emotions, they (and we) consider these viewpoints on the structure of emotions a useful starting point. These various theories have considerable overlap in the basic emotions proposed, even though they were arrived at using diverse methods, including facial expressions of adult humans (Ekman), behaviors of human infants and children (Izard), direct brain stimulation and behavioral responses of non-human animals (Panksepp), and sorting of emotion words by adult humans [39]. Below we consider each emotion for which we included a subscale in the DEQ.

The DEQ is predicated on a discrete, versus a dimensional, approach to emotions. Regarding the ‘debate’ between discrete approaches and dimensional approaches, we believe that both have value. Discrete emotions can be categorized along dimensions, including valence (positivity/negativity), arousal (high/low), and motivational direction (approach/avoid). However, discrete emotions also possess other characteristics that do not fall neatly into dimensions, such as specific action tendencies, subjective feelings, evoking situations, and cognitive appraisals. Whether emotions are viewed as discrete or dimensional, people can still name specific emotion types and having a measure that allows them to distinguish between these different emotion types is useful and important. The emotions measured by the DEQ are listed below.

Anger is often regarded as a negative, high arousal emotion that is associated with approach motivational tendencies [40, 41, 42]. Neurally, anger involves testosterone, substance P (a neuropeptide that acts as a neurotransmitter and neuromodulator), the medial hypothalamus, amygdala, and the periaqueductal gray [6, 43, 44]. The full facial expression of anger involves the muscles of the brow moving inward and downward; the eyes fixed in a hard stare; and the nostrils and wings of the nose expanded [4, 5, 45]. When laypersons sort emotion words into groupings, the Anger cluster includes words like *anger*, *rage*, *irritation*, and *exasperation* [39].

Disgust is also regarded as a negative, high arousal emotion, but it is associated with withdrawal motivational tendencies. Disgust is considered a basic emotion by Ekman and Izard but not by Panksepp, who views disgust as a sensory affect. Shaver et al. [39] found that the two disgust-related words (*disgust*, *revulsion*) included in their emotions list clustered with the anger words, forming a branch along with *contempt*. Thus, if disgust is a basic emotion, researchers may need to devote additional effort to finding words that capture the construct. Neurally, disgust uniquely involves the inferior frontal gyrus/anterior insula relative to other emotions [46]. The full-face expression of disgust involves the brows being drawn down and together and a wrinkled nose. The upper lip is also pulled up and the lower lip is pulled downward. The tongue may be pushed forward out of the mouth [4, 5, 45].

Fear is often regarded as a negative, high arousal emotion that is associated with withdrawal motivational tendencies. Neurally, fear involves corticotrophin releasing factor, adreno-cortico-trophic-hormone, cholecystokinin, central and lateral amygdala, anterior and medial hypothalamus, PAG, and the lower brain stem [6]. The full-face expression of fear involves the eyebrows lifted and slightly pulled together; the eyes opened wide; and the corners of the mouth retracted straight back with the mouth slightly open [4, 5, 45]. When laypersons sort emotion words into groupings, the Fear cluster includes words like *horror*, *alarm*, *terror*, and *fear* [39].

Anxiety is often regarded as a negative, high arousal emotion that is most likely associated with behavioral conflict [47]. Anxiety is not characterized as a "basic" emotion by emotion theorists such as Ekman, Izard, or Panksepp. Similarly, Shaver et al. [39] found that anxiety words (e. g., *anxiety*, *nervousness*, *tenseness*) form a branch within the Fear cluster. However, many areas of research point to the theoretical and empirical importance of distinguishing anxiety from fear. Fear is often evoked by discrete, acute threats, whereas anxiety is evoked by vague, potential threats [48, 49]. Neurally, anxiety is not as well understood as fear, but the brain systems that contribute to anxiety overlap with those that contribute to fear. One key neural difference between fear and anxiety is that the peptide corticotropin-releasing factor (CRF) has a special role in anxiety [49]. The facial expression of anxiety is distinguished from other facial expressions of emotion by eye darts and head swivels that presumably increase the spatial area seen by the eyes [50]. When laypersons sort emotion words, the anxiety branch includes words like *anxiety*, *nervous*, and *tense* [39]. For the purposes of the DEQ, we saw value in attempting to distinguish anxiety from fear, although we expected that these constructs would show some overlap.

Sadness is often conceptualized as a negative, low arousal emotion; it appears to be mostly associated with the approach motivational system. In Panksepp's system, the affect that maps onto sadness is called PANIC/GRIEF. Although this terminology may be surprising to human researchers, PANIC/GRIEF, like human sadness, is a response to loss (particularly the infant animal's separation from the mother), and characterized by distress vocalizations (crying). Neurally, PANIC/GRIEF (i.e., sadness) involves endorphins, corticotrophin releasing factor, cortisol, glutamate, midbrain PAG, medial diencephalon, ventral septal area, preoptic area, bed nucleus of stria terminalis, cingulate gyrus, amygdala, and hypoththalamus [6]. In human neuroimaging studies, sadness uniquely activates middle frontal gyrus and head of the caudate/subgenual anterior cingulate cortex [46]. The full facial expression of sadness involves the inner corners of the brows being drawn obliquely upward and together, with the eyes begin slightly narrowed; the corners of the mouth are pulled downward and the chin may be pushed up and quiver [4, 5]. When laypersons sort emotion words, the Sadness cluster includes words like *suffering*, *sadness*, *depression*, and *disappointment* [39].

Joy or happiness is conceptualized as a positive emotion that could be associated with a variety of intensities of approach motivation; most theoretical perspectives are silent or mixed regarding where joy sits on the arousal dimension. Joy is described as a basic emotion by Ekman and Izard, and joy words form a major cluster in the analysis by Shaver et al. [39]. The conceptually closest emotion in Panksepp's [6] system may be PLAY, which seems similar to the joy posited by other theories. When laypersons sort emotion words, the Joy cluster includes words like *cheerfulness*, *joy*, *enthusiasm*, and *contentment* [39].

Most theories concerned with basic emotions have only carefully considered one or two positive emotions, and we believe that this is unfortunate for emotion science. Of course, theorists, including Ekman [51], have discussed many other positive emotions but research has not delved too far into these discrete positive emotions. One reason for the difficulty in distinguishing discrete positive emotions may be the overly dominant influence of valence on self-reports. In a stark demonstration of this, Pettersson and Turkheimer [13] found that a large first factor, which they refer to as "evaluation" confounds self-reported moods. They created a measure, based on the PANAS, by adding words of opposite meaning but identical valence (for example, *proud* was matched with *humble*, and *determined* was matched with *easy-going*). To assess evaluation, the PANAS items and matched antonyms were rated on how desirable it would be for a person to endorse each item. Separate participants rated the items on how much they felt these words applied to themselves, in general. When the first principal component was extracted from participants' self-endorsement of the items, it correlated *r* = .96 with the evaluation of the items, strongly suggesting that individuals endorsed the items based on valence, rather than semantic content. Similar results have been found in early research [52]. The evaluation component may be equivalent to social desirability [53, 54, 55]. In creating the DEQ, we considered that it may be important to control for this large evaluation component, in order to accurately measure other discrete emotions, particularly positive emotions.

In addition to including a Joy/Happiness subscale (which we suspect would be equivalent to evaluation, or valence) on the DEQ, we aimed to include subscales for two other positive emotional states that have received much theoretical and empirical attention. Broadly speaking, these two states can be thought of as low- and high-approach positive affect. We refer to these subscales as Satisfaction and Desire, respectively. These concepts align with perspectives that examine appetitive or pre-goal positive states as being distinct from consummatory or post-goal positive states [56], or "wanting" as distinct from "liking" [57]. The positive affective states of high-approach (desire) and low-approach (satisfaction) are associated with different neural structures and neurochemicals [6, 56, 57, 58]. Low-approach positive affect involves a small area within the nucleus accumbens and the posterior half of the ventral pallidum, brain regions which are sensitive to opioids and endocannabinoids [57]. High approach positive affect (SEEKING, in Panksepp's system) involves the amygdala, nucleus accumbens, and frontal cortex [59]. The neurotransmitter dopamine, produced in the mesolimbic and mesocortical dopamine circuits, activates these regions. Pregoal, high-approach-motivated positive affect (desire) presumably assists in promoting reward acquisition, whereas postgoal, low-approach-motivated positive affect (satisfaction) presumably assists in promoting reward enjoyment.

The research we review concerning the brain regions, hormones, and neurotransmitters involved in discrete emotions is primarily based on non-human animal evidence, which is typically more invasive and therefore more precise than the research that can be conducted with humans. There is evidence regarding brain regions involved in discrete emotions that is obtained from functional magnetic resonance imaging (fMRI) of humans lying supine while experiencing emotions. A supine posture has been found to reduce certain physiological responses associated with certain emotions [60, 61, 62]. This fMRI evidence presents a more mixed picture as to whether there is any discrete emotions specificity with brain regions. That is, some meta-analyses have suggested there is no support [63], whereas other meta-analyses have suggested there is support for discrete emotions being linked to specific neural circuits [46]. Hamann’s [64] interpretation of this literature is prescient, “… although neuroimaging studies have identified consistent neural correlates associated with basic emotions and other emotion models, they have ruled out simple one-to-one mappings between emotions and brain regions, pointing to the need for more complex, network-based representations of emotion” (p. 458).

Because the DEQ is intended primarily for use in measuring self-reported emotions among lay participants, we were careful to ensure that it was made up of words that are 1) clearly understood by lay English speakers (as opposed to psychological or emotion scientists), and 2) actually used by ordinary individuals in describing their emotional experiences. Instead of developing the DEQ from an existing list of emotion words, we preferred to build the instrument on a foundation of words that are spontaneously used and understood by lay persons. Thus, Study 1 used a bottom-up, open-ended method to collect words used by individuals to name the emotions they experienced in situations that would, according to emotion theory, evoke the emotions of interest: Anger, disgust, fear, anxiety, sadness, joy, desire, and satisfaction.

## Study 1

In Study 1, we aimed to generate a list of words that individuals use to name emotions. Our goal in doing so was to base our preliminary instrument around words that individuals actually use in describing emotional states. To this end, we asked participants to recall emotional events from their own lives, and to name the words that best described the emotions they experienced during these events.

### Method

#### Materials

We created story prompts based on the themes that have been identified in emotional accounts in past research. Smith and Lazarus [65] identified "core relational themes" for anger, guilt, sadness, and fear/anxiety. Shaver et al. [39] analyzed emotional stories and identified the prototypical themes for fear, sadness, anger, joy, and love. The story prompts for Study 1 were based on these themes from past research (Table 1). It is important to note that the prompts avoided the use of emotion words. Instead, they focused on the prototypical characteristics of events that tend to produce the target discrete emotions.

**Table 1:** Study 1: Prompts Used to Elicit Emotional Recall.

Intended Emotion	Story Prompt
Anger	Please remember a SPECIFIC time when someone else was to blame for something bad that happened to you. The person or thing who was at fault harmed you in some way, or prevented you from getting something you wanted. Please think of a negative situation, caused by someone else, in which you experienced an extremely intense emotional response.
Disgust	Please remember a SPECIFIC time when you saw, smelled, touched, or felt something repulsive. The object or person that you came across was nasty, creepy, or sickening. Please think of a negative situation, when you encountered something revolting, in which you experienced an extremely intense emotional response.
Fear	Please remember a SPECIFIC time when you were in danger. You were threatened with harm and you were either uncertain about how to deal with the situation, or felt unable to cope. Please think of a negative situation, when you were faced with being injured or harmed, in which you experienced an extremely intense emotional response.
Anxiety	Please remember a SPECIFIC time when you anticipated something negative happening in the future. You very much wanted something NOT to happen, but you believed that this unpleasant outcome was going to occur soon. Please think of a negative situation, when you were expecting something bad to happen, in which you experienced an extremely intense emotional response.
Sadness	Please remember a SPECIFIC time when you experienced an important loss and you knew that it was impossible to overcome. The person or object that was lost was gone forever, and you knew that nothing could be done to change the situation. Please think of a negative situation, when you lost someone or something important to you, in which you experienced an extremely intense emotional response.
Desire	Please remember a SPECIFIC time when you anticipated something positive happening in the future. You wanted something very much, and you believed that the outcome you hoped for was going to occur soon. Please think of a positive situation, when you were expecting something pleasant to happen, in which you experienced an extremely intense emotional response.
Relaxation	Please remember a SPECIFIC time when something wonderful had just occurred. You got something you wanted very much, or you achieved an important goal, or you had some other experience that was very pleasant. Please think of a positive situation, when something very good had happened to you, in which you experienced an extremely intense emotional response.

#### Participants

Participants were 337 individuals (186 women, 148 men, 1 other, 2 declined to answer) residing in the United States who completed the study online using Amazon's Mechanical Turk. The ethnicities reported by participants were White (261), African American (16), Asian (31), Hispanic/Latino (19), Native American (3), and Other (6). Age ranged from 18 to 73 years (*M* = 34.28, *SD* = 12.78). They were recruited for a study on memories and emotions. This and all other studies in this article were reviewed and approved by The University of New South Wales Human Research Ethics Advisory Panel C: Psychology.

#### Procedure

Because this and the other studies were conducted online, participants did not provide signed consent. They were presented with the consent form and, if they chose to consent, they entered their initials into a box and clicked the option "I consent to participate." The other option was "I do NOT consent to participate." If potential participants selected this option, they were exited from the survey. This was approved by the ethics committee. After giving informed consent, participants saw the following: “On the next screen, you will see instructions regarding the situation we would like you to remember. It is very important to the study that you understand and follow these instructions. Please read carefully!”

Next, participants were randomly assigned to one of the prompts in Table 1. After reading their prompt, they viewed these instructions: “Take a few moments to remember the situation that you thought of. As you remember the incident, re-experience the emotions you felt at that time as strongly as possible. When you have the experience in mind, and the emotions are strong, please go on to the next screen.”

When participants proceeded to the next screen, they read, “At this time, please write down the events of the emotional experience that you remembered. In as much detail as possible, describe what happened, paying particular attention to your thoughts and feelings.” They were given 180 seconds to write about the experience they had remembered.

On the following screen, participants read, “In the space below, write ONE WORD that BEST names the emotion you experienced most strongly during this event.” After giving their open-ended response, participants proceeded to the next screen and read, “In the spaces below, write 4 more words that name the emotion you experienced most strongly during the event you wrote about. If you can't think of 4 words, write as many words as you can, and write 'none' in the other spaces.”

On the following screen, the instructions stated, “Now please imagine that you are in the middle of the situation that you wrote about. You want to tell a close friend how you are feeling. You say, "I am so ___________!" In the space below, please fill in the blank with the word, or words, that you would say to tell a close friend how you feel.” The purpose of this question was to encourage participants to generate vernacular words that they would actually use, in the moment, to name their emotional experiences.

Thus, participants had the opportunity to generate up to 6 words/phrases naming the emotions they experienced in the situation they had remembered.

#### Manipulation checks

To determine whether the emotion manipulation was successful, a judge who was blind to condition coded each story for whether it included each of the eight emotion themes (non-exclusive, S1 Data). Results appear in Table 2. To assess reliability, a second judge coded a random sample of 15% of the stories. Inter-rater reliability was good, Cohen’s Kappa = 0.73.

**Table 2:** Study 1: Story Themes Identified by Coding.

Emotion Prompt	N	Other-blame (Anger)	Contamination (Disgust)	Physical harm (Fear)	Negative anticipation (Anxiety)	Loss (Sadness)	Positive anticipation (Desire)	Post-goal positive (Satisfaction)
Anger	38	**34**	2	4	7	14	1	0
Disgust	46	11	**40**	1	0	4	0	0
Fear	37	20	1	**31**	3	0	0	1
Anxiety	47	12	1	6	**39**	9	1	0
Sadness	46	12	1	0	2	**41**	0	0
High-approach	41	3	0	0	3	4	**26**	9
Low-approach	38	0	0	0	3	0	3	**31**

*Note*. Count of stories in each story-prompt condition that included the emotion theme (non-exclusive). Targeted emotion themes are bolded in each row.

### Results

We performed a count of the words generated for each condition. Different grammatical forms of a word (e. g., anger, angry) were counted as a single word. Table 3 shows the most common words generated in response to each prompt. Words were included in the table if the word was generated at least twice for “Best Word” or “Vernacular,” or at least three times for “Other Words.”

**Table 3:** Study 1: Most Frequently Generated Words for Each Condition.

Type of Prompt	Anger Memory Prompt	Disgust Memory Prompt	Fear Memory Prompt	Anxiety Memory Prompt	Sadness Memory Prompt	Desire Memory Prompt	Satisfaction Memory Prompt
Best Word	• Angry (12) • Frustrated (3)	• Disgust (23)	• Fear (18) • Panic (2) • Scared (2)	• Fear (8) • Anxious (6) • Angry (6) • Sad (5) • Worried (4) • Dread (4)	• Sad (11) • Devastated (5) • Grief (4) • Loss (3)	• Happy (10) • Excited (3) • Ecstatic (3)	• Happy (10) • Love (6) • Proud (5)
Other Words	• Angry (14) • Sad (14) • Hate (6) • Disappointed (6) • Betrayed (5) • Frustrated (5) • Hurt (5) • Upset (5) • Fear (5)	• Sad (10) • Sickened (9) • Disgust (8) • Nausea (8) • Repulsed (8) • Angry (8) • Anxious (7) • Frustrated (6) • Horror (5) • Revolted (5)	• Scared (14)	• Sad (14) • Fear (11) • Anxious (10) • Angry (9) • Nervous (6) • Frustrated (5) • Love (5)	• Sad (21) • Angry (13) • Loss (7) • Lonely (7) • Lost (6) • Hurt (5) • Helpless (5) • Empty (5) • Despair (5)	• Happy (20) • Excited (11) • Joy (9) • Anticipation (8) • Nervous (6) • Anxiety (5) • Hopeful (5)	• Happy (15) • Excited (12) • Joy (11) • Ecstatic (6) • Proud (6) • Nervous (6) • Thankful (5)
Vernacular	• Pissed off (10) • Angry (6) • Mad (3)	• Disgust (14) • Grossed out (8) • Sickened (3)	• Angry (12) • Scared (7) • Fear (6) • Sad (5) • Worried (5) • Anxious (3) • Confused (3) • Nervous (3) • Panic (3)	• Scared (4) • Worried (4) • Stressed out (3) • Frustrated (3) • Pissed off (3)	• Sad (12) • Hurt (4) • Lost (4) • Powerless (3)	• Excited (13) • Happy (8)	• Happy (10) • Excited (8) • Blessed (4)

*Note*: Numerals indicate number of times word was generated.

From this list, we created a preliminary word list for the emotion instrument, with six items per emotion category. This preliminary list included the most frequently generated words for each emotion prompt which overlapped minimally with words generated in response to the other emotion prompts. For the categories for which participants did not generate at least six common words, we supplemented the list with items from Shaver et al. (1987) that fell near the participant-generated words in a cluster analysis, and that were rated as being good exemplars of emotion (rated at least 3.00 on a 4-point scale where 1 = I definitely *would not* call this an emotion and 4 = I definitely *would* call this an emotion) [38].

## Study 2

For Study 2, participants recalled an emotional experience (similar to Study 1), but instead of generating emotion words, they rated the extent to which they had experienced emotions during the experience, using the preliminary emotion scale developed in Study 1.

### Method

#### Participants

Participants were 244 individuals (139 female, 104 male, 1 other) who completed the study on Amazon’s Mechanical Turk. Their reported ethnicities were White (197), African American (15), Asian (14), Hispanic/Latino (11), Native American (1), and Other (5). Age ranged from 18 to 68 years (*M* = 32.91; *SD* = 11.02). Of these participants, 25 were in the love condition, which was dropped from the ANOVA (in all studies) because the love words never loaded on their own factor. Despite including additional positive affect words, a unique factor for Love words did not emerge in the factor analysis. The word “*love”* and its synonyms had high loadings on the first factor (Happiness). Therefore, we dropped the Love subscale from the DEQ. However, the love words were used in all factor analyses because all participants in all conditions responded to them as well as the other words.

#### Procedure

The method was identical to Study 1, except that after participants finished writing about the emotional experience they had recalled, they rated, on a 7-point scale (1 = Not at all and 7 = An extreme amount), the extent to which they had experienced the emotions, using the items on the preliminary scale. The emotion items were presented in a random order. We assumed all participants followed the instructions because each one wrote about an emotional experience.

### Results

Using Statistica Version 12 (StatSoft, Inc.), the data (S2 Data) were subjected to a factor analysis using maximum likelihood with varimax (raw) rotation; cases with missing values were rejected on pairwise basis (Table 4). Initially, the number of factors allowed was 8, because we predicted that 8 factors would result. This analysis yielded five factors with Eigenvalues greater than 1.00. The first factor had an Eigenvalue of 24.92, and all of the positively valenced words loaded negatively on this factor. The second factor had an Eigenvalue of 5.58, and all of the *fear* and *anxiety* words loaded positively on this factor (except “shock,” which had a loading of 0.44). The third factor had an Eigenvalue of 2.14, and all of the *anger* words loaded positively on this. The fourth factor had an Eigenvalue of 2.43, and all of the *sad* words loaded on this factor. The fifth factor had an Eigenvalue of 1.58, and all of the *disgust* words loaded on this factor.

**Table 4:** Study 2: Exploratory Factor Analysis Results.

Preliminary Word List	Factor 1 Happiness/ Evaluation	Factor 2 Fear/ Anxiety	Factor 3 Anger	Factor 4 Sadness	Factor 5 Disgust
**Happy**	**-0.90**	-0.21	-0.20	-0.23	-0.14
**Enjoyment**	**-0.90**	-0.23	-0.19	-0.20	-0.12
**Enthusiasm**	**-0.89**	-0.17	-0.19	-0.22	-0.15
Satisfaction	-0.89	-0.23	-0.19	-0.22	-0.09
**Liking**	**-0.88**	-0.21	-0.18	-0.13	-0.11
Pleasure	-0.87	-0.22	-0.18	-0.21	-0.10
Thankful	-0.86	-0.19	-0.21	-0.12	-0.14
Optimism	-0.83	-0.18	-0.17	-0.17	-0.11
Excitement	-0.83	-0.10	-0.21	-0.25	-0.16
Adoration	-0.79	-0.24	-0.18	0.02	-0.11
Hope	-0.78	-0.05	-0.23	-0.18	-0.14
Pride	-0.78	-0.16	-0.13	-0.17	-0.10
Contentment	-0.78	-0.25	-0.13	-0.12	-0.07
Eager	-0.76	-0.05	-0.13	-0.21	-0.12
Attraction	-0.75	-0.19	-0.15	-0.02	-0.10
Affection	-0.72	-0.26	-0.10	0.18	-0.13
Anticipation	-0.64	0.21	-0.18	0.02	0.10
Love	-0.63	-0.23	-0.15	-0.28	-0.20
Caring	-0.62	-0.15	-0.23	0.27	-0.17
**Nervous**	0.05	**0.82**	0.11	0.01	0.03
**Fear**	0.21	**0.81**	0.21	0.20	0.12
**Scared**	0.16	**0.78**	0.13	0.29	0.14
**Worry**	0.29	**0.77**	0.22	0.21	0.11
**Anxiety**	0.23	**0.77**	0.21	0.18	0.06
**Tense**	0.30	**0.75**	0.28	0.15	0.11
**Panic**	0.25	**0.74**	0.33	0.18	0.21
Stressed out	0.35	0.68	0.35	0.24	0.06
Dread	0.36	0.64	0.27	0.32	0.20
**Terror**	0.18	**0.61**	0.17	0.23	0.37
Alarm	0.33	0.58	0.21	0.13	0.33
Shock	0.20	0.44	0.30	0.24	0.39
**Mad**	0.37	0.29	**0.79**	0.24	0.12
**Rage**	0.28	0.33	**0.77**	0.18	0.19
**Anger**	0.37	0.33	**0.77**	0.28	0.10
**Pissed off**	0.39	0.27	**0.74**	0.21	0.19
Hate	0.20	0.29	0.63	0.13	0.29
Frustration	0.44	0.38	0.55	0.25	0.14
**Grief**	0.25	0.28	0.15	**0.76**	0.12
**Sad**	0.38	0.29	0.27	**0.73**	0.06
**Empty**	0.21	0.20	0.14	**0.73**	0.17
**Depression**	0.26	0.27	0.30	**0.73**	0.06
Lonely	0.20	0.27	0.22	0.72	0.02
Devastation	0.26	0.35	0.35	0.65	0.19
**Grossed out**	0.27	0.10	0.15	-0.02	**0.77**
**Revulsion**	0.26	0.26	0.32	0.18	**0.68**
**Sickened**	0.34	0.33	0.39	0.27	**0.60**
**Nausea**	0.22	0.34	0.09	0.23	**0.57**
Disgust	0.35	0.20	0.52	0.10	0.52
Horror	0.27	0.50	0.25	0.21	0.51

*Note*: Items used for preliminary subscales are bold.

These results suggested that the negative emotion words differentiated into specific emotions, with the exception of fear and anxiety. However, the positive emotion words on our preliminary scale loaded on the large first factor, which, based on past research [13], is equivalent to evaluation/valence.

Composite variables (subscales) were created by taking the mean of the four items with the highest loadings on each factor and small cross-loadings on other factors (shown in bold in Table 4). Subscales were not created for the positive affect items, because these did not load on separate factors. To examine whether the subscales were sensitive to our emotion manipulation, one-way analyses of variance (ANOVA) were conducted with the emotion manipulations as independent variables and the mean ratings for each emotion category as the dependent measures (Table 5). These results showed that these “best four” items were elevated by the expected manipulations, with the exception of the anger subscale and the anxiety subscale. For the anger subscale, the anger prompt elevated ratings significantly more than any except the fear prompt. The increase in the mean for the anger prompt compared to the fear prompt was not quite statistically significant (*p* = .06). For the anxiety subscale, the anxiety prompt elevated ratings more than any prompts except the anger and fear prompts. Means for the anger prompt did not differ from those for the anxiety prompt (*p* = .73). Furthermore, means for the fear prompt elevated ratings on the anxiety subscale more than any other prompt, including anxiety (*p* = .03). These results suggested that our preliminary scale discriminated between all negative emotions except anxiety and, to some extent, anger. Based on these results, we conducted Study 3 with a more controlled emotion manipulation and additional positive emotion words.

**Table 5:** Study 2: Mean Responses on Preliminary Subscales in Response to Emotional Recall Prompts.

Subscale	Anger prompt	Disgust prompt	Fear prompt	Anxiety prompt	Sadness prompt	Desire prompt	Satisfaction prompt	One-Way ANOVA
Anger subscale	**5.36a (1.67)**	2.97b (1.81)	4.53a (2.27)	4.18b (1.88)	3.95b (2.07)	1.22b (0.69)	1.23b (0.87)	F (6, 212) = 29.02, p < .001
Disgust subscale	3.02b (1.64)	**4.98a (1.69)**	3.63b (1.90)	2.62b (1.41)	2.59b (1.28)	1.25b (0.39)	1.39b (0.87)	F (6, 212) = 27.73 p < .001
Fear subscale	3.86b (1.95)	3.11b (1.64)	**5.76a (1.26)**	3.96b (1.78)	3.16b (1.83)	1.78b (0.84)	1.78b (1.25)	F (6, 212) = 26.53, p < .001
Anxiety subscale	5.02a (1.48)	3.73b (1.61)	6.04c (0.96)	**5.16a (1.59)**	3.82b (1.63)	3.19b (1.64)	2.30b (1.65)	F (6, 212) = 23.02, p < .001
Sadness subscale	3.26b (1.91)	2.37b (1.32)	3.59b (2.29)	3.42b (1.62)	**4.89a (1.54)**	1.33b (0.71)	1.47b (1.13)	F (6, 212) = 21.80, p < .001

Means and SDs (in parentheses) in the target condition are bolded in each row. Within rows, different subscripts differ from the mean in the target condition at p < .05 using Fisher’s LSD test.

## Study 3

For Study 3, we wanted to examine how well the scale discriminated among discrete emotions using a different manipulation of emotion. As the coding results from Study 1 showed, participants’ responses to the prompts were very rich: many of the stories that participants wrote included multiple emotion themes (Table 2). For this current study, guided imagery was used to elicit a more specific emotional response.

In addition, because the positive items on the preliminary scale all loaded on the same factor, we added additional positive items in an attempt to identify items that would discriminate between discrete positive affects. We based these additional items on theoretical and empirical distinctions between high- and low-approach positive affect [6, 57]. For high-approach positive affect, the additional items were *desire*, *craving*, *wanting*, *longing* and *need*. For low-approach positive affect, the additional items were *peace*, *easygoing*, *chilled out*, *savoring*, *calm*, and *relaxation*.

### Method

#### Participants

Participants were 439 individuals (253 women, 184 men, 1 other, 1 declined to answer) recruited from MTurk. Their reported ethnicities were White (352), African American (26), Asian (24), Hispanic/Latino (29), Native American (3), and Other (3). Age ranged from 18 to 68 years (*M* = 32.21; *SD* = 10.57). Of these participants, 55 were in the love condition, which was dropped from the ANOVA because of the reasons stated previously. At the end of the questionnaires, seven participants failed to correctly answer a multiple-choice question indicating the gist of their assigned story. For two pairs of stories, the themes were similar and thus the response options were similar.

#### Materials

The stimuli were composite stories created from typical memories submitted by participants in Study 1 and Study 2. Two scenarios were created for each emotion category (Table 6).

**Table 6:** Study 3: Scenarios Used to Elicit Emotion.

Anger scenario 1	A guy I lived with was a real big douche. He thought he was hot stuff and his little pick-up was a speed demon. One time my car was parked in a gravel parking lot and I noticed it had rock dings all over it. I had many times witnessed him doing donuts in the gravel and slinging rocks everywhere. I knew it was him, and when I asked him about it, he denied knowing anything about it. I was furious because now my car’s paint had rock dings all over it and his dumb self was responsible.
Anger scenario 2	My boss kept telling me I could do more, and a position in another department opened up. She kept pushing me to apply for the job, and made me feel certain I would get it since she knew the hiring manager. I applied and went through the whole interview process. Later the hiring manager called me in the office and told me that I did not receive the job. She said my boss had told her I would not be a good fit. I was so upset. I quit that day and I was so mad because I felt like we were actually friends. She completely stabbed me in the back. I have never been treated like that by anyone and could not understand why she would do something like that to me.
Disgust scenario 1	The house next door was up for rent. I was outside and this guy came to look at the house and asked me a few questions. He had this giant growth or giant zit or something on his bottom lip. It was huge and looked like it was full of pus or something. He just kept talking to me, and I tried not to look at it. It was so gross. It ranks with the most disgusting things I have seen in my life. I tried to keep my distance from him, but he kept getting in my personal space while talking to me.
Disgust scenario 2	I was watching TV one day, minding my own business, and suddenly this repulsive smell fills my living room. I looked around to see what it was, and my dog had had diarrhea on my floor. Not only that, but there was also blood in the stool. I promptly got up to clean the mess, but the smell was almost unbearable, and it was also impossible to wipe up without getting it on my hands.
Fear scenario 1	It was a very bad storm and a tornado had touched down near my house. I was very scared that it would hit the house and I would be injured. I spent the entire night curled up in the bathtub with pillows and blankets. I remember listening for how loud the wind was getting outside and my heart felt like it was beating out of my chest. I just kept hoping the storm would pass quickly and the tornado’s path would not come on to my street. I was praying that the people whose houses had been hit were safe.
Fear scenario 2	I was living by myself at the time and I woke up in the middle of the night. I heard a creaking noise and it seemed to be getting a little louder. It sounded like someone walking quietly on the old wooden floorboards. I just stayed in bed and didn’t turn on the lights, listening intently. I wished I had kept the handgun my father had given me for protection, but I had traded it for a shotgun that was put away in the closet. Suddenly, I heard a great crash and the door opening, and I jumped out of bed.
Anxiety scenario 1	About three months before, I had taken the bar exam in order to become a practicing attorney. That day, the results of the exam were to be posted for everyone to see. This put me in the most devastatingly negative mood that I had undergone in a long time. I paced around wondering what my future would be until the moment the results were posted. Then I had to take a very long walk before I could even think of looking. I was a nervous wreck thinking about what sort of job I would have to get if I failed, and how much money I would have to spend to retake it. I was losing it before I even knew the results.
Anxiety scenario 2	I knew that I was going to fail two of my college classes in my final semester, and I was dreading the outcome. I knew that failing them would drop my GPA and I might even have to drop out. I had major assignments pending in both classes, which I could not get myself to complete even in the face of these consequences. I instead hoped that somehow it wouldn't happen, and agonized over the prospect, but did nothing to change the outlook.
Sadness scenario 1	I lost my cat Mittens. I had her for 14 years, and she was a big part of my life. Mittens was the first pet I had ever had that was my own. One day she got outside and I waited at the window watching for her. I actually thought I would see her again so I just waited every day. I remember that I cried almost every night. My nose even bled a few times. It seems silly to be so sad about a cat but she was all I had left of my childhood. I still needed her. I had a few dreams about her and the last one I had I was able to hold her one last time. I miss her very much.
Sadness scenario 2	`I lost my father about 3 years ago. He was in the hospital by himself. He had been in and out of the hospital so much that I thought this time would be no different. But it was different. He died. I couldn't believe it. I still can't believe it. I am still devastated by it. I miss him so much. I hate that people have to die. It doesn't seem fair. There is deep, deep hurt and pain. There is a sense of needing help very strongly. I want my dad back.
Desire scenario 1	When I was young, I wanted this particular skateboard deck really bad, so my dad purchased the deck. However, a condition of me actually mounting and using the deck was that I had to wait until Christmas. I knew where it was stored and I would sometimes look at it and touch it, but never removed the shrink wrap. I would imagine skating on it and I couldn’t wait until Christmas.
Desire scenario 2	I remember waiting to find out whether I got offered a new job. I had done the initial group interview and a follow-up interview with an administrator for this position. I felt very good about both interviews and was extremely hopeful that it would culminate in a job offer. Three weeks went by without any word, so my hopes began to fall. Then, I got a request from HR for my list of references. So my hopes rose again.
Satisfaction scenario 1	I remember being in high school and coming home from school one day. It was my birthday and my mom asked me what I wanted. I told her I wanted a Playstation 2. It had just come out and was pretty expensive. I thought I was going to have to save my lunch money for a year to buy one. Later in the afternoon, my mom asked if I wanted to go to Target. I went straight to the electronics department and looked at the new Playstation. My mom came up behind me, with the guy in charge, and he took one out. She told me she was going to buy it for my birthday. I remember the guy saying I was a very lucky boy. That was one of the happiest days of my life.
Satisfaction scenario 2	My dad called me one day to tell me that he was sending me something to help with my photography and had put something else in the box he was sending as well. A few days later a box from Amazon showed up, addressed to me. When I opened it I found a new telephoto lens he knew I had been saving money for and the "something else" was a note that said, "How to shoot airshows: Get rid of that cheap piece of shit lens and use this instead." The emotions? They were intense.

#### Procedure

The method was identical to Study 2, except that, instead of being asked to recall an emotional memory, participants read, “On the next screen, you will read about the situation we would like you to imagine. Please read carefully! It is very important to this study. The story you will read is a composite of several similar stories written by different people. These are the kinds of stories people often write when they're asked to remember an emotional experience. As you read the story, please put yourself in the place of the person who is describing the experience. Try to imagine, as vividly as possible, how you would feel if this situation were happening to you.” Participants were then randomly assigned to read one of the scenarios from Table 6. Participants then rated the emotions they experienced while imagining the story, on our emotion scale. Finally, they answered a multiple choice question that assessed which story they read.

### Results

The data (S3 Data) were subjected to the same factor analysis parameters as used in Study 2. Our original conceptual prediction was eight factors or emotion subscales. However, Study 2 revealed fear and anxiety to be highly related and to load on the same factor. Thus, in Study 3, we set the factor analysis to have seven factors (Table 7). The first factor had an Eigenvalue of 25.02 and all of the positive items loaded negatively on this factor. The second factor had an Eigenvalue of 8.65 and all of the fear and anxiety items loaded on this factor. The third factor had an Eigenvalue of 3.71 and all of the anger items loaded on this factor. The fourth factor had an Eigenvalue of 2.73 and all of the sadness items loaded on this factor. The fifth factor had an Eigenvalue of 1.99 and all of the desire items loaded on this factor. The sixth factor had an Eigenvalue of 1.47 and all of the disgust items loaded on this factor. The seventh factor had an Eigenvalue of 0.94 and all of the relaxation items loaded on this factor; however these items also cross-loaded on the first factor. Although we began this research thinking that low approach positive affect might be best captured by a word like “satisfaction,” the factor analysis did not reveal that “satisfaction” loaded separately from other positive words. Because the words that did load (somewhat) separately from happiness were associated with and included the word “relaxation,” we labelled this last factor “relaxation.”

**Table 7:** Study 3: Factor Loadings.

Item	Factor 1 Happiness/ Evaluation	Factor 2 Fear/ Anxiety	Factor 3 Anger	Factor 4 Sadness	Factor 5 Desire	Factor 6 Disgust	Factor 7 Relaxation
**Liking**	**-0.89**	-0.25	-0.17	-0.06	0.07	-0.09	-0.01
Pleasure	-0.89	-0.28	-0.18	-0.10	0.03	-0.11	-0.02
**Enjoyment**	**-0.88**	-0.28	-0.18	-0.12	0.04	-0.13	-0.01
**Happy**	**-0.88**	-0.30	-0.19	-0.13	0.05	-0.14	-0.02
Enthusiasm	-0.87	-0.23	-0.14	-0.19	0.18	-0.12	0.05
**Satisfaction**	**-0.85**	-0.23	-0.16	-0.06	0.05	-0.06	-0.06
Contentment	-0.83	-0.18	-0.12	-0.03	0.02	-0.04	-0.15
Thankful	-0.83	-0.25	-0.19	0.01	0.06	-0.11	0.03
Excitement	-0.82	-0.13	-0.09	-0.22	0.21	-0.13	0.12
Adoration	-0.81	-0.17	-0.10	0.09	0.08	-0.04	-0.02
Affection	-0.80	-0.21	-0.15	0.21	0.00	-0.08	0.00
Pride	-0.78	-0.14	-0.05	-0.06	0.14	-0.02	-0.05
Optimism	-0.76	-0.12	-0.17	-0.12	0.19	-0.10	-0.03
Peace	-0.76	-0.23	-0.16	0.00	-0.08	-0.07	-0.41
**Easy-going**	-0.74	-0.22	-0.14	-0.06	-0.04	-0.05	**-0.39**
Love	-0.72	-0.24	-0.16	0.44	0.06	-0.08	0.08
Attraction	-0.70	-0.15	-0.12	-0.04	0.30	0.00	0.00
Savoring	-0.70	-0.10	-0.04	-0.07	0.20	0.03	-0.17
**Relaxation**	-0.68	-0.17	-0.12	0.03	-0.07	-0.03	**-0.52**
Hope	-0.66	0.07	-0.17	-0.07	0.29	-0.12	-0.06
Caring	-0.65	-0.07	-0.25	0.44	0.02	-0.07	-0.03
Eager	-0.63	0.01	-0.01	-0.21	0.45	-0.09	0.10
**Calm**	-0.59	-0.23	-0.14	0.02	0.02	-0.01	**-0.54**
**Chilled Out**	-0.59	-0.11	-0.09	-0.02	-0.04	-0.01	**-0.45**
**Fear**	0.23	**0.86**	0.07	0.11	0.01	0.00	0.02
**Scared**	0.19	**0.86**	0.08	0.17	-0.05	0.11	0.05
**Panic**	0.26	**0.83**	0.20	0.09	0.00	0.08	0.03
**Terror**	0.12	**0.82**	0.12	0.07	-0.07	0.12	0.03
**Nervous**	0.27	**0.81**	0.12	0.05	0.16	0.01	0.04
**Worry**	0.35	**0.80**	0.17	0.15	0.08	0.03	0.03
**Dread**	0.32	**0.78**	0.15	0.19	0.01	0.12	0.03
Tense	0.35	0.69	0.34	0.02	0.14	0.13	0.07
**Anxiety**	0.35	**0.68**	0.22	0.02	0.17	0.08	0.06
Alarm	0.28	0.65	0.34	0.08	-0.06	0.26	0.05
Stressed	0.41	0.65	0.36	0.06	0.09	0.11	0.05
Horror	0.10	0.63	0.33	0.14	-0.09	0.34	0.03
**Anger**	0.26	0.20	**0.86**	0.14	0.02	0.14	0.05
**Mad**	0.25	0.19	**0.86**	0.14	0.01	0.11	0.03
**Pissed-Off**	0.26	0.17	**0.85**	0.11	0.02	0.16	0.04
**Rage**	0.14	0.22	**0.84**	0.08	-0.01	0.16	0.01
Hate	0.09	0.17	0.76	0.01	-0.03	0.15	-0.04
Frustration	0.31	0.35	0.62	0.16	0.24	0.17	0.07
Shock	0.05	0.28	0.54	0.19	-0.13	0.35	0.18
**Lonely**	0.13	0.35	0.21	**0.65**	0.19	-0.04	-0.09
**Sad**	0.24	0.39	0.36	**0.62**	0.12	0.03	0.02
**Grief**	0.17	0.39	0.40	**0.61**	0.07	0.12	0.04
**Empty**	0.16	0.35	0.33	**0.60**	0.20	0.04	-0.02
Depression	0.19	0.41	0.40	0.56	0.17	0.06	0.00
Devastation	0.18	0.42	0.47	0.53	0.02	0.11	0.08
**Longing**	-0.29	0.08	0.02	0.44	**0.68**	-0.06	-0.06
**Wanting**	-0.35	0.08	0.06	0.22	**0.65**	-0.10	0.05
**Craving**	-0.45	0.03	0.10	0.09	**0.62**	-0.03	0.01
**Desire**	-0.55	-0.08	-0.06	0.03	**0.58**	-0.06	0.00
Need	-0.10	0.36	0.16	0.42	0.56	0.00	0.07
Anticipation	-0.46	0.24	-0.06	-0.24	0.45	-0.13	0.05
**Grossed-Out**	0.13	0.04	0.11	-0.08	-0.06	**0.82**	-0.04
**Revulsion**	0.17	0.19	0.35	0.03	-0.07	**0.77**	0.03
Disgust	0.24	0.08	0.50	-0.05	-0.04	0.69	0.00
**Nausea**	0.17	0.42	0.19	0.16	0.02	**0.66**	0.02
**Sickened**	0.22	0.37	0.40	0.18	-0.02	**0.60**	0.06

*Note*: Items in bold were used to create subscales, selected for large loadings on the target factor with small cross-loadings.

Composite variables (subscales) were created by taking the mean of the four items with the highest loadings on each factor (shown in bold in Table 7). For all of the negative affect subscales, these words were identical to those used in Study 2. To examine whether these subscales were sensitive to our emotion manipulations, one-way ANOVAs were conducted with the emotion scenarios as independent variables and the average ratings for each subscale as the dependent measures (Table 8). These results showed that average ratings on the subscales were elevated by the intended manipulations, with a few exceptions.

**Table 8:** Study 3: Mean Responses on Subscales in Response to Emotional Scenarios.

Subscale	Anger 1 scenario	Anger 2 scenario	Disg 1 scenario	Disg 2 scenario	Fear 1 scenario	Fear 2 scenario	Anx 1 scenario	Anx 2 scenario	Sad 1 scenario	Sad 2 scenario	Desire 1 scenario	Desire 2 scenario	Relax 1 scenario	Relax 2 scenario	One-Way ANOVA
Anger subscale	**5.69a (1.25)**	**5.14a (1.53)**	2.30b (1.50)	2.97b (1.72)	1.97b (1.41)	3.58b (2.00)	2.59b (1.58)	3.24b (1.57)	2.31b (1.36)	4.00b (1.90)	1.36b (0.76)	1.73b (1.08)	1.02b (0.07)	1.78b (1.51)	F (13, 364) = 23.87, p < .001
Disgust subscale	2.88c (1.23)	2.87c (1.43)	**4.13a (1.46)**	**5.22b (1.50)**	1.92c (1.16)	2.20c (1.21)	2.44c (1.07)	2.76c (1.60)	1.90c (1.04)	2.43c (1.26)	1.27c (0.82)	1.29c (0.46)	1.04c (0.14)	1.41c (0.98)	F (13, 364) = 25.35, p < .001
Fear subscale	2.58c (1.59)	2.40c (1.41)	1.93c (0.90)	2.88c (1.36)	**4.96a (1.55)**	**5.23b (1.56)**	4.43a (1.51)	4.27c (1.75)	3.30c (1.95)	2.98c (1.41)	1.26c (0.70)	2.21c (1.07)	1.06c (0.13)	1.33c (0.76)	F (13, 364) = 28.59, p < .001
Anxiety subscale	3.19c (1.25)	3.39c (1.41)	2.84c (1.06)	3.61c (1.47)	4.95b (1.37)	5.13ab (1.52)	**5.66a (1.12)**	**5.37ab (1.80)**	4.24c (1.87)	3.77b (1.33)	1.77c (0.93)	3.86c (1.29)	1.47c (0.62)	1.56c (0.96)	F (13, 364) = 29.83, p < .001
Sadness subscale	2.42b (1.30)	2.91b (1.27)	1.58b (0.90)	1.97b (1.00)	2.66b (1.35)	2.59b (1.31)	2.37b (1.13)	3.74b (1.43)	**4.71a (1.69)**	**5.23a (1.29)**	1.29b (0.80)	1.55b (0.90)	1.21b (0.57)	1.47b (1.07)	F (13, 364) = 30.93., p < .001
Desire subscale	1.63c (0.92)	2.02c (1.04)	1.18c (0.66)	1.26c (0.74)	1.58c (0.92)	1.91c (1.16)	2.71c (1.48)	2.64c (1.28)	3.43b (1.25)	3.44b (1.55)	**5.16a (1.29)**	**3.78b (1.41)**	3.88b (1.66)	2.17c (1.18)	F (13, 364) = 24.72, p < .001
Relax subscale	1.41c (0.91)	1.34c (0.95)	1.63c (0.93)	1.30c (0.57)	1.37c (0.76)	1.25c (0.42)	1.27c (0.59)	1.33c (0.51)	1.24c (0.40)	1.33c (0.66)	2.41a (1.13)	1.88c (1.20)	**3.26a (1.52)**	**2.85a (1.19)**	F (13, 364) = 14.30, p < .001

Means and SDs (in parentheses) in the target conditions are bolded in each row. Within rows, means/SDs with different subscripts differ from the means in the target conditions at p < .05 using Fischer’s LSD test.

For the fear subscale, only fear scenario 2 elevated ratings more than any other manipulation. For the anxiety subscale, ratings following anxiety scenario 1 did not significantly differ from fear scenario 2, and ratings following anxiety scenario 2 did not differ significantly from fear scenario 1 or 2. For the desire subscale, only desire scenario 1 elevated ratings more than any other scenario. For the relax subscale, ratings following relax scenario 2 differed only marginally (*p* = .07) from ratings following desire scenario 1.

These results showed that each of the subscales was sensitive to manipulations of the intended discrete emotion, although some of the emotional scenarios were more effective than others. The only exception was the anxiety subscale, which was sensitive to both anxiety and fear scenarios.

## Study 4

In Study 4, we wanted to assess whether our emotion instrument was sensitive to a different manipulation of emotion. Study 2 used autobiographical recall, whereas Study 3 used guided imagery. For Study 4, we chose a pictorial manipulation, because photographs are often used to manipulate affect in psychological research.

### Method

#### Participants

Participants were 511 individuals who completed the study through MTurk. Three participants exited the study after completing the baseline questionnaire. Seventeen participants were excluded for failing the manipulation check question (they did not correctly identify the content of the two picture sets, baseline and emotional, they had viewed), leaving 491 individuals (290 women, 193 men, 3 other) for the analyses. Their reported ethnicities were White (366), African American (32), Asian (55), Hispanic/Latino (21), Native American (2), and Other (10). Age ranged from 18 to 79 years (*M* = 32.19; *SD* = 11.02). Five participants did not respond to these demographics questions.

#### Materials

We created seven sets of photographs, intended to evoke the seven emotions measured by the instrument. Each set included five photographs. The emotional sets were anger (militants burning the American flag) [43, 66], disgust (dirty toilets and rotten food), fear (dangerous animals and weapons), anxiety (people taking tests or being questioned by police), sadness (people crying) [67], desire (delicious-looking desserts) [68], and relaxation (beaches). We also created a neutral set, which was made up of pictures of rocks [69]. Although some of the photographs were photos from the International Affective Picture System [70] (disgust IAPS numbers 7359, 7380, 9300, 9320, 9570; fear IAPS numbers 1050, 1220, 1300, 6230, 6350; sad IAPS numbers 2205, 2800, 9050, 9210, 9220), many were selected from the Internet and had been used in our previous research [42, 65, 66, 67, 68].

#### Procedure

Before beginning the task, participants read these instructions, “On the next few screens, you will see several pictures. Please view each picture for the entire time it is presented. Each picture will be shown for 4 seconds, and then the display will automatically advance to the next picture. As you view the set of pictures, imagine yourself experiencing the events that the pictures show. As vividly as you can, try to place yourself in the scene and imagine that what is shown in the photos is actually occurring to you right now. After you view several pictures, you will rate how the set of pictures made you feel while viewing them.” We included these instructions to manipulate emotion as well as possible, to better understand the self-reported subjective experience of emotions. Other researchers have used a different (and more minimal) set of instructions for picture viewing manipulations, but we are unsure why they have chosen to use the instructions they use. We suspect that their choice reflects their primary research agenda of focusing more on psychophysiological responses to individual pictures (averaged over many of the same valence and arousal level) than on an extensive measurement of self-reported subjective experience of emotions.

To familiarize them with the task, participants then viewed the neutral set of rock pictures. They rated their emotions while viewing the set on the items active, alert, attentive, jittery, hostile, guilty, inspired, ashamed, upset and strong. Next participants read the picture viewing instructions again.

Participants were then randomly assigned to one set of pictures. They viewed each photograph for 4 seconds. After viewing all the pictures of their assigned set, they rated how they felt during viewing on the DEQ (that is, an instrument made up of the best 4 items from each of the 8 factors).

Participants then completed the Attitudes Toward Emotions scale (results of which are not of interest for this study and are not reported), demographic questions, and attention check questions that asked them to describe the two picture sets they had viewed.

Composite variables for the data (S4 Data) were created by taking the mean of each subscale, using the same items as in Study 3.

### Results

To examine whether these subscales were sensitive to our emotional picture manipulation, one-way analyses of variance (ANOVA) were conducted with the picture sets as independent variables and the average ratings on each emotion subscale as the dependent measures (Table 9). These results showed that ratings on the discrete emotion subscales were elevated by the expected picture sets, with minor exceptions. Although ratings on the fear subscale were elevated more by the fear pictures than by the anxiety pictures, ratings on the anxiety subscale were elevated equally by fear and anxiety pictures. Also, ratings on the desire subscale were only marginally higher for desire pictures compared to relaxation pictures (p = .06). Reliability for all subscales was high (Cronbach’s *α*s > 0.80, Table 10).

**Table 9:** Study 4: Mean Responses on Subscales in Response to Emotional Photographs.

Subscale	Anger photos	Disgust photos	Fear photos	Anxiety photos	Sadness photos	Desire photos	Relaxation photos	One-Way ANOVA
Anger subscale	**3.54a (1.90)**	2.81b (1.64)	2.96b (1.48)	2.66b (1.66)	2.23b (1.40)	1.18b (0.51)	1.23b (0.74)	F (7, 483) = 24.18, p < .001
Disgust subscale	2.88b (1.69)	**4.69a (1.42)**	3.12b (1.33)	1.77b (0.97)	2.19b (1.30)	1.24b (0.77)	1.23b (0.69)	F (7, 483) = 61.40, p < .001
Fear subscale	2.63b (1.54)	2.22b (1.27)	**3.52a (1.59)**	2.68b (1.56)	2.50b (1.60)	1.12b (0.39)	1.24b (0.70)	F (7, 483) = 27.50, p < .001
Anxiety subscale	3.04b (1.60)	2.77b (1.29)	3.70a (1.54)	**3.68a (1.78)**	2.99b (1.56)	1.21b (0.50)	1.29b (0.69)	F (7, 483) = 39.33, p < .001
Sadness subscale	2.42b (1.23)	2.28b (1.23)	2.12b (1.27)	2.49b (1.32)	**3.28a (1.46)**	1.39b (0.69)	1.48b (0.86)	F (7, 483) = 15.59, p < .001
Desire subscale	1.23b (0.65)	1.17b (0.53)	1.29b (0.78)	1.70b (0.96)	1.78b (1.11)	**4.20a (1.69)**	3.86a (1.70)	F (7, 483) = 72.92, p < .001
Relaxation subscale	1.44b (0.89)	1.39b (0.74)	1.29b (0.71)	1.64b (0.86)	1.59b (0.93)	3.28b (1.43)	**4.71a (1.75)**	F (7, 483) = 74.73, p < .001

Means and SDs (in parentheses) in the target conditions are bolded in each row. Within rows, means/SDs with different subscripts differ from the means in the target conditions at p < .05 using Fisher’s LSD test.

**Table 10:** Subscale Reliabilities,

Subscale	Study 2	Study 3	Study 4
Anger subscale (anger, mad, pissed off, rage)	0.97	0.96	0.94
Disgust subscale (grossed out, revulsion, sickened, nausea)	0.88	0.89	0.92
Fear subscale (terror, scared, fear, panic)	0.92	0.94	0.93
Anxiety subscale (worry, anxiety, dread, nervous)	0.90	0.93	0.93
Sadness subscale (lonely, grief, sad, empty)	0.85	0.89	0.82
Desire subscale (wanting, craving, longing, desire)	*	0.86	0.93
Relaxation subscale (calm, relaxation, chilled out, easygoing)	*	0.91	0.93
Happiness subscale (happy, enjoyment, satisfaction, liking)	*	0.97	0.96

*Note*: Numbers represent Cronbach’s *α*;

* indicates that the subscale was not included in this study.

Given the strong predictions derived from past theory and research, we thought the use of controls for family-wise error rate was unnecessary. However, when Bonferroni corrections were applied to the ANOVAs (.05/7 = .0071), the *p* value needs to be less than .007 to be acceptable. In all cases, the *p* values were acceptable. For Studies 2, 3, and 4, all *p* values for the ANOVAs were less than .001. Study 1 did not use ANOVA.

## General Discussion

The results of four studies support the validity of the DEQ, a discrete emotions questionnaire (S1 Appendix). Study 1 was intended to generate a starting base of emotion words that are used by laypeople to name the emotions they experience in various situations. In Study 2, these words, supplemented as needed with emotion words from Shaver et al. [39], were used to create a preliminary emotion measure. Participants recalled emotional experiences, as in Study 1, and indicated their endorsement of these words. Exploratory factor analysis revealed 5 factors which we called positive emotion, fear/anxiety, anger, sadness, and disgust. In Study 3, additional positive words were included to better capture discrete positive emotions, and participants rated their endorsement of these words after guided imagery of emotional scenarios. Exploratory factor analysis revealed 7 factors which we called happiness, fear/anxiety, anger, sadness, desire, disgust, and relaxation. Results of Study 3 were conceptually replicated in Study 4 which revealed that the ratings on the DEQ subscales were influenced in predictable ways by a different manipulation of emotion states.

The DEQ consists of the 4 items with the highest loadings on each of these factors (8 from the fear/anxiety factor) and small cross-loadings on the other factors. Moreover, the internal consistency of each subscale is more than adequate and experimental manipulations of emotion influence the emotion subscales in the predicted manner in all studies. The results from the ANOVAs clearly indicate that the Discrete Emotions Questionnaire is sensitive at assessing discrete emotional responses to discrete emotional events. The exploratory factor analyses also supported the existence of discrete emotions, even though they were unable to take into account the experimental manipulation of emotions. It is important to note that, in the validation studies for other affect scales such as the PANAS, emotions were not manipulated; rather participants were asked to endorse the items while in a neutral setting, presumably not experiencing strong emotion [7]. In contrast, participants in the current studies were assigned to experience one of 8 emotions, adding an important source of variance to their ratings. Past research has revealed that inducing emotions changes the factor structure of affect scales[11, 12].

Although we could have conducted a study similar to the validation studies for the PANAS, by asking participants to respond to the DEQ by indicating how they feel in a non-emotional context, we believe that such a study would be uninformative. The DEQ is intended to measure state emotions, so in a non-emotional context, responses to the negative emotion subscales of the DEQ (anger, disgust, fear, anxiety, and sadness) should be near the floor for most individuals. Past research and the current studies show that persons report fairly high positive emotions (happiness, relaxation, and desire) at rest, either because people feel fairly positive at baseline or because of ‘evaluation’ or social desirability [13]. The goal in creating the DEQ was to create an instrument that would be sensitive to state emotions. In our view, whether the instrument has good factor structure in the absence of emotion-inducing situations is not informative. The ANOVA results of the current research show that the subscales of the DEQ appropriately detect manipulated state emotions.

These studies showed that the DEQ is sensitive to several different manipulations of emotion: autobiographical recall, guided imagery, and pictorial stimuli. Scores on the target subscales were elevated significantly more than scores on the other subscales by these stimuli. The only exception was the Fear and Anxiety subscales: these items loaded on a single fear/anxiety factor, however, participants endorsed them differently in response to emotional manipulations. Fear manipulations elevated both Fear and Anxiety ratings, whereas anxiety manipulations elevated only Anxiety ratings, but not Fear ratings. We surmise that the subjective experiences of both fear and anxiety include the uncertainty and anticipation of an unpleasant future expressed in words such as “worry” and “dread,” however, only the experience of fear also includes the expectation of physical harm expressed in words like “terror” and “fear” [65]. Given that the Fear and Anxiety items behave differently in this way, we chose to retain both subscales in the DEQ. We based our design of the studies and inclusion of both anxiety and fear on existing theories and research that have revealed differences between anxiety and fear, as reviewed earlier. Although anxiety and fear “typically loaded together” in the factor analyses, when we consider responses to the emotional inductions, we find that situations that evoke fear cause increases in DEQ fear and anxiety, but situations that cause anxiety only cause increase in DEQ anxiety but not fear. If both fear and anxiety were not included in the DEQ, then future research might miss these important differences in outcomes between fear and anxiety situations. Thus, we included the two subscales in the DEQ.

The DEQ can be used as a relatively short measure of the “basic” emotions of anger, disgust, fear, sadness, and happiness, plus the theoretically interesting emotions of anxiety, desire (high approach), and relaxation (low approach positive). Although these are certainly not the only discrete emotions, they cover the broad categories considered basic and important by a number of emotion theorists [3, 4, 5, 39].

We have developed a measure of emotion that is sensitive to discrete emotional states, and maintains a decent overall factor structure when capturing dynamic emotional states. Yet, it may not be necessary to use the entire DEQ when measuring emotion. In our view, researchers should use emotion theory, past research, and the purposes of his/her research question to determine whether to use the entire DEQ or only a selection of its subscales. In some cases, the use of a few subscales or a single subscale of the DEQ might be sufficient. Using the full scale may be beneficial when a researcher wants to examine whether participants are experiencing mixed emotions, or when a manipulation may cause one emotion for some participants and a different emotion for other participants. In other cases, the participant fatigue brought on by completing many items might outweigh the benefits of measuring more emotions than those directly of interest.

We note that although we have validated a coherent measure comprising eight subscales that are sensitive to situational changes in emotional states, we have not exhausted full possibilities of the DEQ. Rather we view this as a work in progress that may be contributed to over time. For instance, researchers might find that they are interested in specific emotions not currently included in the DEQ. We would suggest that in these cases the DEQ, as specified here, could be useful for assisting those researchers to construct measures of additional emotions (by modelling the approach used in the DEQ), and to demonstrate the discriminant validity of those measures (by showing they do not overlap with other measures in the DEQ). Overtime, additional emotions may be added to, and validated as part of, the DEQ.

In the end, we hope that the DEQ will be of use to researchers interested in measuring self-reported discrete emotions associated with emotional states. As reviewed earlier, much past research has used a flawed measure presumably because it appeared to have good measurement properties, was published in the field’s top empirical journal, and was so widely used. We believe our understanding of psychological and behavioral processes will be better understood by using a measure such as the DEQ that more accurately captures emotional responses to events. Indeed, we have already reported the results of three experiments that revealed the DEQ to be more sensitive than the PANAS at detecting self-reported emotions to discrete events [71]. Emotions are vital psychological constructs that need to be measured as well as possible so that research can fully understand their impact on psychological processes.

## Supporting Information

S1 Appendix The Discrete Emotions Questionnaire. DEQ with instructions. (DOCX)

S1 Data Data file for Study 1. Generate words following emotion-evoking prompts. (XLSX)

S2 Data Data file for Study 2. Rate words following emotion-evoking prompts. (XLSX)

S3 Data Data file for Study 3. Rate words following emotion-evoking scenarios. (XLSX)

S4 Data Data file for Study 4. Rate words following emotion-evoking photographs. (XLSX)

## References

[1] Darwin C. The descent of man and selection in relation to sex New York: Appleton & Co.; 1872

[2] James W. What is an emotion?. Mind. 1884 4 1(34):188–205.

[3] Ekman P. An argument for basic emotions. Cogn Emot. 1992;6(3–4):169–200.

[4] Izard CE. The face of emotion New York: Appleton-Century-Crofts; 1971.

[5] Izard CE. The psychology of emotions New York: Plenum Press; 1991.

[6] Panksepp J. Affective neuroscience: The foundations of human and animal emotions Oxford: Oxford University Press; 1998.

[7] Watson D, Clark LA, Tellegen A. Development and validation of brief measures of positive and negative affect–The PANAS scales. J Pers Soc Psychol. 1988;54(6):1063–70. 3397865 10.1037//0022-3514.54.6.1063

[8] Baron RM, Kenny DA. The moderator mediation variable distinction in social psychological research–Conceptual, strategic, and statistical considerations. J Pers Soc Psychol. 1986;51(6):1173–82. 3806354 10.1037//0022-3514.51.6.1173

[9] Harmon-Jones E, Greenberg J, Solomon S, al. e The effects of mortality salience on intergroup bias between minimal groups. Eur J Soc Psychol. 1996;26(4):677–81.

[10] Watson D, Wiese D, Vaidya J, Tellegen A. The two general activation systems of affect: Structural findings, evolutionary considerations, and psychobiological evidence. J Pers Soc Psychol. 1999;76(5):820–38.

[11] Egloff B, Schmukle SC, Burns LR, Kohlmann CW, Hock M. Facets of dynamic positive affect: Differentiating joy, interest, and activation in the Positive and Negative Affect Schedule (PANAS). J Pers Soc Psychol. 2003;85(3):528–40. 14498788 10.1037/0022-3514.85.3.528

[12] Harmon-Jones E, Harmon-Jones C, Abramson L, Peterson CK. PANAS Positive Activation Is Associated With Anger. Emotion. 2009;9(2):183–96. 10.1037/a0014959 19348531

[13] Pettersson E, Turkheimer E. Approach temperament, anger, and evaluation: Resolving a paradox. J Pers Soc Psychol. 2013;105(2):285–300. 10.1037/a0033046 23773039

[14] Harmon-Jones E, Harmon-Jones C. On the relationship of trait PANAS positive activation and trait anger: Evidence of a suppressor relationship. J Res Pers. 2010;44(1):120–3.

[15] Mayer JD, Gaschke YN. The experience and meta-experience of mood. J Pers Soc Psychol. 1988 7;55(1):102 3418484 10.1037//0022-3514.55.1.102

[16] Bradley MM, Lang PJ. Measuring emotion–the self-assessment mannequin and the semantic differential. J Behav Ther Exp Psychiatry. 1994;25(1):49–59. 7962581 10.1016/0005-7916(94)90063-9

[17] McNair P. M., Lorr M., & Droppleman L. F. POMS manual (2nd ed.). San Diego: Educational and Industrial Testing Service; 1981.

[18] Zuckerman M, Lubin B, Robins S. Validation of the multiple affect adjective check list in clinical situations. J Consult Psychol. 1965 12 31;29(6):594 5846133 10.1037/h0022750

[19] Gross JJ, Levenson RW. Emotional suppression–Physiology, self-report, and expressive behavior. J Pers Soc Psychol. 1993;64(6):970–86. 8326473 10.1037//0022-3514.64.6.970

[20] Gulliksen H. Theory of mental tests New York, NY: John Wiley & Sons; 1950.

[21] Epstein S. Aggregation and beyond–Some basic issues on the prediction of behavior. J Personal. 1983;51(3):360–92. 10.1111/j.1467-6494.1983.tb00338.x 28497598

[22] Crede M, Harms P, Niehorster S, Gaye-Valentine A. An Evaluation of the Consequences of Using Short Measures of the Big Five Personality Traits. J Pers Soc Psychol. 2012;102(4):874–88. 10.1037/a0027403 22352328

[23] Burisch M. Approaches to personality-inventory construction–A comparison of merits. Am Psychol. 1984;39(3):214–27.

[24] Burisch M. Test length and validity revisited. Eur J Pers. 1997;11(4):303–15.

[25] Buss AH, Durkee A. An inventory for assessing different kinds of hostility. J Consult Psychol. 1957 8;21(4):343 13463189 10.1037/h0046900

[26] Cohen TR, Wolf ST, Panter AT, Insko CA. Introducing the GASP Scale: A New Measure of Guilt and Shame Proneness. J Pers Soc Psychol. 2011;100(5):947–66. 10.1037/a0022641 21517196

[27] Haidt J, McCauley C, Rozin P. Individual differences in sensitivity to disgust–A scale sampling 7 domains of disgust elicitors. Pers Individ Dif. 1994;16(5):701–13.

[28] Lyubomirsky S, Lepper HS. A measure of subjective happiness: Preliminary reliability and construct validation. Soc Indic Res. 1999;46(2):137–55.

[29] McCullough ME, Emmons RA, Tsang JA. The grateful disposition: a conceptual and empirical topography. J Pers Soc Psychol. 2002 1;82(1):112 11811629 10.1037//0022-3514.82.1.112

[30] Modigliani A. Embarrassment and embarrassability. Sociometry. 1968 9 1:313–26. 5681346

[31] Shiota MN, Keltner D, John OP. Positive emotion dispositions differentially associated with Big Five personality and attachment style. J Posit Psychol. 2006 4 1;1(2):61–71.

[32] Smith RH, Parrott WG, Diener EF, Hoyle RH, Kim SH. Dispositional envy. Pers Soc Psychol Bull. 1999 8 1;25(8):1007–20.

[33] Tangney JP, Dearing RL, Wagner PE, Gramzow R. The test of self-conscious affect–3 (TOSCA-3) Fairfax, VA: George Mason University; 2000.

[34] Tracy JL, Robins RW. The psychological structure of pride: a tale of two facets. J Pers Soc Psychol. 2007 3;92(3):506 17352606 10.1037/0022-3514.92.3.506

[35] Spielberger CD, Gorssuch RL, Lushene PR, Vagg PR, Jacobs GA. Manual for the state-trait anxiety inventory Consulting Psychologists Press, Inc; 1983.

[36] Marschall D, Sanftner J, Tangney JP. The state shame and guilt scale Fairfax, VA: George Mason University; 1994.

[37] Zinner LR, Brodish AB, Devine PG, Harmon-Jones E. Anger and asymmetrical frontal cortical activity: Evidence for an anger-withdrawal relationship. Cogn Emot. 2008;22(6):1081–93. 10.1080/02699930701622961 PMC10191169 37200986

[38] Ekman P, Friesen WV. Constants across cultures in face and emotion. J Pers Soc Psychol. 1971;17(2):124 5542557 10.1037/h0030377

[39] Shaver P, Schwartz J, Kirson D, O'Connor C. Emotion knowledge: further exploration of a prototype approach. J Pers Soc Psychol. 1987 6;52(6):1061 3598857 10.1037//0022-3514.52.6.1061

[40] Berkowitz L, Harmon-Jones E. Toward an understanding of the determinants of anger. Emotion. 2004;4(2):107–30. 15222847 10.1037/1528-3542.4.2.107

[41] Carver CS, Harmon-Jones E. Anger is an approach-related affect: Evidence and implications. Psychol Bull. 2009;135 (2):183–204. 10.1037/a0013965 19254075

[42] Harmon-Jones E, Harmon-Jones C, Price TF. What is Approach Motivation? Emot Rev. 2013;5(3):291–5.

[43] Peterson CK, Harmon-Jones E. Anger and testosterone: Evidence that situationally-induced anger relates to situationally-induced testosterone. Emotion. 2012 Oct;12(5):899 10.1037/a0025300 21910539

[44] Siegel A. Neurobiology of aggression and rage New York: Taylor & Francis US; 2004.

[45] Ekman P, Friesen WV. Unmasking the face Englewood Cliffs: Prentice-Hall; 1975.

[46] Vytal K, Hamann S. Neuroimaging support for discrete neural correlates of basic emotions: a voxel-based meta-analysis. J Cogn Neurosci. 2010 Dec;22(12):2864–85. 10.1162/jocn.2009.21366 19929758

[47] Gray JA, McNaughton N. The neuropsychology of anxiety Oxford: Oxford University Press; 2000.

[48] Blanchard RJ, Blanchard DC, editors. Advances in the study of aggression Amsterdam: Elsevier; 2013.

[49] Davis M, Walker DL, Miles L, Grillon C. Phasic vs Sustained Fear in Rats and Humans: Role of the Extended Amygdala in Fear vs Anxiety. Neuropsychopharmacology. 2010;35(1):105–35. 10.1038/npp.2009.109 19693004 PMC2795099

[50] Perkins AM, Inchley-Mort SL, Pickering AD, Corr PJ, Burgess AP. A facial expression for anxiety. J Pers Soc Psychol. 2012 May;102(5):910 10.1037/a0026825 22229459

[51] Ekman P. Emotion revealed New York: Henry Holt and Co; 2003.

[52] Edwards AL. Trait and evaluative consistency in self-description. Educ Psychol Meas. 1969;29(4):737.

[53] Meehl PE, Hathaway SR. The K factor as a suppressor variable in the Minnesota Multiphasic Personality Inventory. J Appl Psychol. 1946;30(5):525 20282179 10.1037/h0053634

[54] Paulhus DL. Two-component models of socially desirable responding. J Pers Soc Psychol. 1984 3;46(3):598.

[55] Wiggins JS. Convergences among stylistic response measures from objective personality tests. Educ Psychol Meas. 24(3), 551–562. 1964.

[56] Knutson B, Wimmer GE. Splitting the difference. Annals of the New York Academy of Sciences. 2007 5 1;1104(1):54–69. 17416922 10.1196/annals.1390.020

[57] Berridge KC. The debate over dopamine's role in reward: the case for incentive salience. Psychopharmacology. 2007;191(3):391–431. 17072591 10.1007/s00213-006-0578-x

[58] Harmon-Jones E. Unilateral right-hand contractions cause contralateral alpha power suppression and approach motivational affective experience. Psychophysiology. 2006;43(6):598–603. 17076816 10.1111/j.1469-8986.2006.00465.x

[59] Panksepp J, Biven L. The archaeology of mind: neuroevolutionary origins of human emotions (Norton series on interpersonal neurobiology) New York: WW Norton & Company; 2012.

[60] Harmon-Jones E, Gable PA, Price TF. Leaning embodies desire: Evidence that leaning forward increases relative left frontal cortical activation to appetitive stimuli. Biol Psychol. 2011;87(2):311–3. 10.1016/j.biopsycho.2011.03.009 21440032

[61] Harmon-Jones E, Peterson CK. Supine Body Position Reduces Neural Response to Anger Evocation. Psychological Science. 2009;20(10):1209–10. 10.1111/j.1467-9280.2009.02416.x 19656336

[62] Price TF, Dieckman LW, Harmon-Jones E. Embodying approach motivation: Body posture influences startle eyeblink and event-related potential responses to appetitive stimuli. Biol Psychol. 2012 7 31;90(3):211–7. 10.1016/j.biopsycho.2012.04.001 22522185

[63] Lindquist KA, Wager TD, Kober H, Bliss-Moreau E, Barrett LF. The brain basis of emotion: a meta-analytic review. Behav Brain Sci. 2012 6 1;35(03):121–43. 22617651 10.1017/S0140525X11000446 PMC4329228

[64] Hamann S. Mapping discrete and dimensional emotions onto the brain: controversies and consensus. Trends Cogn Sci. 2012;16(9):458–66. 10.1016/j.tics.2012.07.006 22890089

[65] Smith CA, Lazarus RS. Appraisal components, core relational themes, and the emotions. Cogn Emot. 1993 5 1;7(3–4):233–69.

[66] Harmon-Jones E, Harmon-Jones C, Amodio DM, Gable PA. Attitudes Toward Emotions. J Pers Soc Psychol. 2011;101(6):1332–50. 10.1037/a0024951 21843012

[67] Gable P, Harmon-Jones E. The Blues Broaden, but the Nasty Narrows: Attentional Consequences of Negative Affects Low and High in Motivational Intensity. Trends Cogn Sci. 2010;21(2):211–5. 10.1177/0956797609359622 20424047

[68] Gable P, Harmon-Jones E. Relative left frontal activation to appetitive stimuli: Considering the role of individual differences. Psychophysiology. 2008;45(2):275–8. 18047483 10.1111/j.1469-8986.2007.00627.x

[69] Gable PA, Harmon-Jones E. Late Positive Potential to Appetitive Stimuli and Local Attentional Bias. Emotion. 2010;10(3):441–6. 10.1037/a0018425 20515232

[70] Lang PJ, Bradley MM, Cuthbert BN. International affective picture system (IAPS): Affective ratings of pictures and instruction manual. Technical Report A-8. University of Florida, Gainesville, FL; 2008.

[71] Harmon-Jones C, Bastian B, Harmon-Jones E. Detecting transient emotional responses with improved instructions and measures. Emotion; in press. 10.1037/emo0000216 27685155

