# Supplementary material for: The Discrete Emotions Questionnaire: A New Tool for Measuring State Self-Reported Emotions
Source: PLoS One. 2016 Aug 8;11(8):e0159915. doi: 10.1371/journal.pone.0159915 (PMC4976910; doi:10.1371/journal.pone.0159915)
Supplement: S1 Appendix — (DOCX) [file pone.0159915.s001.docx]

**The Discrete Emotions Questionnaire**

Please indicate your response using the scale provided.

While *(undergoing the emotional experience, e. g., viewing the photographs, reading the story, etc.)* to what extent did you experience these emotions?

| 1 | 2 | 3 | 4 | 5 | 6 | 7 |
| --- | --- | --- | --- | --- | --- | --- |
| Not at all | Slightly | Somewhat | Moderately | Quite a bit | Very much | An extreme amount |

| Anger (Ag) | Scared (F) |
| --- | --- |
| Wanting (Dr) | Mad (Ag) |
| Dread (Ax) | Satisfaction (H) |
| Sad (S) | Sickened (Dg) |
| Easygoing (R) | Empty (S) |
| Grossed out (Dg) | Craving (Dr) |
| Happy (H) | Panic (F) |
| Terror (F) | Longing (Dr) |
| Rage (Ag) | Calm (R) |
| Grief (S) | Fear (F) |
| Nausea (Dg) | Relaxation (R) |
| Anxiety (Ax) | Revulsion (Dg) |
| Chilled out (R) | Worry (Ax) |
| Desire (Dr) | Enjoyment (H) |
| Nervous (Ax) | Pissed off (Ag) |
| Lonely (S) | Liking (H) |

Ag = Anger items, Dg = Disgust items, F = Fear items, Ax = Anxiety items, S = Sadness items, Dr = Desire items, R = Relaxation items, H = Happiness items.
